# New Insights into the Mechanisms of Chinese Herbal Products on Diabetes: A Focus on the “Bacteria-Mucosal Immunity-Inflammation-Diabetes” Axis

**DOI:** 10.1155/2017/1813086

**Published:** 2017-10-15

**Authors:** Zezheng Gao, Qingwei Li, Xuemin Wu, Xuemin Zhao, Linhua Zhao, Xiaolin Tong

**Affiliations:** ^1^Department of Endocrinology, Guang'anmen Hospital, China Academy of Chinese Medical Sciences, Beijing 100054, China; ^2^Shenzhen Hospital, Guangzhou University of Chinese Medicine, Guangzhou 518034, China; ^3^Laboratory of Molecular Biology, Guang'anmen Hospital, China Academy of Chinese Medical Sciences, Beijing 100053, China

## Abstract

Diabetes, especially type 2, has been rapidly increasing all over the world. Although many drugs have been developed and used to treat diabetes, side effects and long-term efficacy are of great challenge. Therefore, natural health product and dietary supplements have been of increasing interest alternatively. In this regard, Chinese herbs and herbal products have been considered a rich resource of product development. Although increasing evidence has been produced from various scientific studies, the mechanisms of action are lacking. Here, we have proposed that many herbal monomers and formulae improve glucose homeostasis and diabetes through the BMID axis; B represents gut microbiota, M means mucosal immunity, I represents inflammation, and D represents diabetes. Chinese herbs have been traditionally used to treat diabetes, with minimal side and toxic effects. Here, we reviewed monomers such as berberine, ginsenoside, *M. charantia* extract, and curcumin and herbal formulae such as Gegen Qinlian Decoction, Danggui Liuhuang Decoction, and Huanglian Wendan Decoction. This review was intended to provide new perspectives and strategies for future diabetes research and product.

## 1. Introduction

In July 2015, the International Diabetes Federation (IDF) released the seventh edition of “IDF diabetes map,” which showed that China had approximately 1.096 million diabetes cases in 2015, ranking the highest in the world. According to the current development trend, the number of diabetes cases in China is projected to reach 150.7 million in 2040. The incidence of diabetes is estimated to increase by 69% in developing countries and 20% in developed countries from 2010 to 2030. Thus, schemes in preventing and treating diabetes are warranted. Currently, Chinese herbs for the prevention and treatment of diabetes and its minimal complications are considered advantageous, and a large number of evidence-based clinical studies have confirmed these effects [1–7].

Modern medical technology provides a new way and direction for the prevention and treatment of diabetes. Chinese herbs have been historically and traditionally used in the treatment of diabetes, which dates back to the Qin dynasty (221 to 207 BC). Globalization and progress in medical science rejuvenate the ancient Chinese herbs, and an increasing number of studies have shown that various specific monomers and Chinese formulae can be used in the prevention and treatment of diabetes through the “Bacteria-Mucosal Immunity-Inflammation-Diabetes” axis (BMID axis). We retrieved the literature and screened out more than 40 relevant Chinese herbs and its derivatives that have been used to treat diabetes regulating multiple targets. We have chosen some of them which have been widely studied and characterized, including monomers and prescriptions. The specific monomers include berberine, *M. charantia* extract, ginsenoside, and curcumin, and prescriptions include Gegen Qinlian Decoction (GGQLD), Danggui Liuhuang Decoction (DGLHD), and Huanglian Wendan Decoction (HLWDD). Here, we attempt to explore the possible mechanisms of action of these in diabetic treatment from the perspective of immunology and provide potentially novel therapeutic strategies that may improve clinical treatment on diabetes.

## 2. New Insights into Diabetes: “Bacteria-Mucosal Immunity-Inflammation-Diabetes” Axis

The gut microbiota represents a microbial community located in the intestine, composed of over a trillion microorganisms with hundreds of species, which play an important role in digestion and intestinal mucosal immunity. There is increasing evidence that confirms that the changes in gut microbiota are associated with insulin resistance and diabetes.

### 2.1. Diabetes Is Associated with Imbalance of Gut Microbiota

In mice and humans, there are two main bacterial phyla, Bacteroidetes and Firmicutes, which are found in the gut through metagenomic analysis [8]. The normal mice keep a relative balance among these two bacterial phyla, but the bacterial phylum Firmicutes is increased in obese mice observably [9]. A study has shown that a high-fat diet (HFD) can reduce the number of *Bifidobacterium* species (spp.), resulting in the development of endotoxemia and diabetes; an oligosaccharide-rich diet increases the number of *Bifidobacterium* species (spp.) and subsequently reduces the level of inflammation and improves glucose intolerance [10]. The metagenomic analysis revealed that patients with type 2 diabetes had moderate levels of gut microbiota dysbiosis, characterized by the decrease in the abundance of some universal butyrate-producing bacteria and the increase in the abundance of some bacteria which reduce sulfate and antioxidant stress [11]. A human study showed that the number of *Faecalibacterium prausnitzii*, which was associated with the production of short-chain fatty acids and butyrate, is increased [12]. Gut microbiota of diabetic patients and mice changed significantly, indicating that reversing this change may reduce the incidence of insulin resistance and diabetes.

### 2.2. Disorder of Mucosal Immunity Leads to Diabetes

Gut microbiota affects insulin resistance and type 2 diabetes mellitus (T2DM) by altering the intestinal epithelial barrier and intestinal mucosal immunity. The main function of the intestinal epithelial barrier is to limit intestinal contents such as water, chyme, and gut microbiota and also to regulate immune responses. The epithelial barrier needs a continuous epithelial cell layer, and the tight junctions are the major characteristic. The tight junctions consist of a complex network of transmembrane proteins, cytoplasmic proteins, and regulatory proteins. There are two pathways: “pore” and “leak”. The “pore” pathway is a high-capacity, size-selective, and charge-selective route, and the “leak” pathway refers to a low-capacity pathway that has limited selectivity [13]. In HFD-induced mice, intestinal bacteria or bacterial products cause the elevation of tumor necrosis factor (TNF) and interleukin- (IL-) 13. These inflammatory factors increase transcription and activity of the myosin light-chain kinase (MLCK) and IL-13-dependent claudin-2 and subsequently increase permeability of “pore” and “leak” pathways and the pass of lipopolysaccharide (LPS) [14–16]. This change leads to an increase in chronic inflammation in the liver, fat, and other tissues and other metabolic diseases. In addition to the intestinal bacterial products, the gut microbiota can also directly pass the intestinal barrier and translocate to the pancreatic lymph nodes, activate NOD-like receptors 2 (NOD2), and contribute to diabetes [17].

The intestinal mucosal immunity is the most important line of defense against intestinal infection through the functions of goblet cells, intestinal epithelial cells (IEC), innate lymphoid cells (ILC), and other rapid responsive immune cells, such as macrophages and neutrophils. The goblet cells and IEC can produce antimicrobial peptides (AMPs) and mucin to prevent pathogens from penetrating the intestine [18]. IEC can secrete anti-inflammatory mediators such as IL-25, IL-33, and transforming growth factor-*β* (TGF-*β*). These mediators can influence micro-associated molecular patterns (MAMPs) by binding to Toll-like receptors 5 (TLR5) and NOD2. ILC inhibits the body's chronic low-level inflammation through the secretion of IL-22, IL-17A, IL-17F, and so forth [19]. HFD can reduce the diversity and alter the distribution balance of gut microbiota and thus reduce the production of mucin and other antimicrobial factors. Invasive bacteria and the associated products alter the intestinal mucosal immunity, and that contributes to the development of T2DM [20].

### 2.3. Inflammation Affects Diabetes through Multiple Pathways

The alternation of intestinal mucosal immunity and increased production of inflammatory factors have been considered to be linked to the development of T2DM. The pathophysiological processes are proposed to mainly include three pathways: (1) The nuclear factor kappa-B (NF-*κ*B) pathway are activated by the inhibitor of nuclear factor kappa-B kinase (IKK) and proinflammatory cytokines such as TNF-*α*, IL-1, and IL-6. The activated IKK and proinflammatory cytokines phosphorylate the inhibitor of NF-*κ*B (I*κ*B). When I*κ*B is phosphorylated, I*κ*B and NF-*κ*B are dissociated, resulting in NF-*κ*B degradation. Then, NF-*κ*B enters the nucleus, thereby mediating the expression of a variety of inflammatory genes [21, 22]. (2) IKK regulates insulin receptor substrate serine/threonine phosphorylation, interfering with normal tyrosine phosphorylation and weakening the insulin signal transduction. IKK is currently considered the link between inflammation and insulin resistance (IR). TNF-*α* and free fatty acid (FFA) activate Jun N-terminal kinase (JNK) and product insulin receptor substrate number 307 serine, which interferes with the insulin signal transduction via the IR/IRS/PI3K pathway. (3) The SOCS family of the cytokine signaling factor (SOCS) pathway mediates cytokine-induced IR. SOCS-1, SOCS-3, and SOCS-6 are involved, which predominantly inhibit the phosphorylation of IRS1 and IRS2 tyrosine residues and accelerate the degradation of IRS1 and IRS2 [23, 24].

### 2.4. “Bacteria-Mucosal Immunity-Inflammation-Diabetes” Axis

It has been shown that gut microbiota affects the intestinal epithelial barrier and intestinal mucosal immunity, alters the level of inflammation, and influences insulin resistance and diabetes. This potential pathogenesis of diabetes is referred to as the “BMID” axis, “B” represents gut microbiota, “M” represents mucosal immunity, “I” represents inflammation, and of course, “D” represents diabetes. This axis is like a “line” to string most antidiabetic agents together and may provide new perspectives and strategies for future research on diabetes and the development of hypoglycemic drugs.

## 3. Herbal Monomers

Herbal monomers are major effective constituents of Chinese herbs. Studies on monomers are increasing in recent years, because they have specific molecular structure and are easier to be used in mechanism research and observing effective targets of Chinese herbs. Here, we screened out five representative monomers to find out their different functions based on the BMID axis. They have been widely researched and applied in treating diabetes for a long time.

### 3.1. Berberine

**Figure 1:**
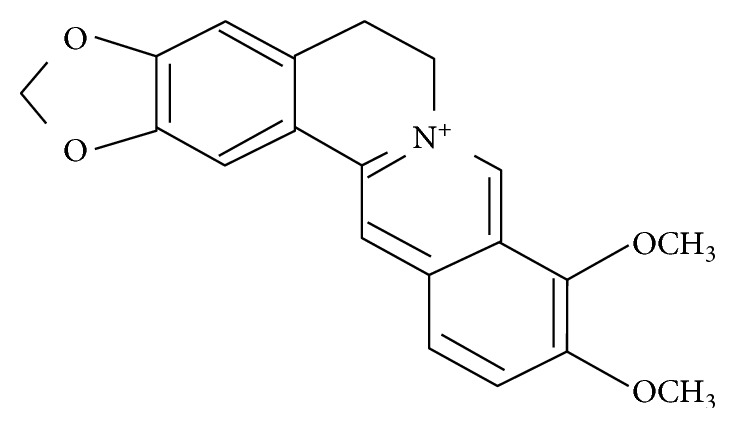
Molecular structure of berberine.

*Rhizoma coptidis* has been used for centuries in traditional Chinese medicine (TCM) as an antipyretic and alexipharmacons, and its main active component is berberine (BBR, Figure 1) [25]. BBR is an isoquinoline and found in many plants of the berberidaceae family. Recent studies have shown that BBR and its derivatives possess a variety of disease-fighting activities, they regulate the immune system, inhibit inflammation, and reduce insulin resistance [26, 27], and they have an anticancer effect likewise; it was reported that BBR inhibits the progression of pancreatic, colon, and breast cancer [28–31].

#### 3.1.1. Effect of BBR on Diabetes

Insulin resistance (IR) is a metabolic state in which insulin inefficiently regulates the tissue and cell for their uptake and utilization of glucose. Nod-like receptor family pyrin domain containing 3 (NLRP3) contributes to obesity-induced inflammation and insulin resistance [32]. A recent study showed that BBR inhibited saturated fatty acid palmitate- (PA-) induced activation of NLRP3 and release of interleukin-1*β* (IL-1*β*) in macrophages by activating AMPK-dependent autophagy, thus reducing inflammation and insulin resistance [26]. An animal study showed that BBR reduced blood TNF-*α*, IL-6, and MCP-1 levels of JNK and IKK*β* phosphorylation in obese mice fed with a high-fat diet, as well as indicated that BBR improves insulin resistance possibly through inhibiting the activation of macrophages in adipose tissue [33].

#### 3.1.2. Effect of BBR on Gut Microbiota and Intestinal Mucosal Immunity

Intestinal barrier integrity and immune tolerance are associated with the pathogenesis of diabetes. Defects in the integrity of the mucosal barriers and leakage of the gut microbiome can contribute to the low-grade inflammation of tissues, which is well known to be associated with glucose metabolism in the muscle, liver, and adipose tissue and causes glucose intolerance and T2DM [17, 34]. Recent studies have shown that BBR imparts beneficial effects on the immune cells of the intestinal immune system and influences the expression of intestinal immune factors. It also inhibits the expression of IL-1*β*, IL-4, IL-10, MIF, and TNF-*α* mRNA and reduces the low-grade inflammation [35].

Intestinal microflora is an important factor in mediating the development of obesity-related metabolic disorders (including type 2 diabetes). The current results suggest that BBR can regulate the intestinal microflora, restore the intestinal barrier, reduce metabolic endotoxemia and systemic inflammation, and improve gut peptide levels in high-fat diet-fed rats; it indicates that BBR is possibly an effective agent for the treatment of obesity and diabetes [36]. A study showed that BBR improved metabolic disorders induced by high-fat diets by modulating the gut-intestinal-brain axis, including changes in the distribution and diversity of microbes, elevation of serum glucagon-like peptide-1 and neuropeptide Y, decrease in orexin A, and upregulation of glucagon-like peptide-1 receptor mRNA [37].

#### 3.1.3. BBR Reduces Inflammatory Response in Diabetes Mellitus

The anti-inflammatory activity of BBR has been observed in in vitro and in vivo studies. In immunocytes (macrophages) [26, 38], cultured metabolic cells (adipocytes and hepatocytes) [39, 40], or pancreatic *β* cells [41], BBR treatment reduces the production of TNF-*α*, IL-6, IL-1*β*, inducible nitric oxide synthase (iNOS), cyclooxygenase-2 (COX-2), matrix metalloprotease 9 (MMP9), monocyte chemoattractant protein-1 (MCP-1), and CRP and haptoglobin (HP) increases the transcription of Nrf2-targeted antioxidative genes [*NADPH quinone oxidoreductase*-1 (*NQO-1*), *heme oxygenase-*1 (*HO-1*)] [37–41]. The insulin-sensitizing effect of HepG2 cells is closely related to its anti-inflammatory activity. BBR administration significantly decreased serine phosphorylation and increased tyrosine phosphorylation of IRS in HepG2 cells, which improved insulin signaling and thus in turn ameliorated insulin resistance [40]. BBR inhibited inflammation, ameliorated insulin resistance, and reduced the production of proinflammatory cytokines such as IL-6, IL-17, TNF-*α*, and interferon-*γ* (IFN*γ*) in NOD mice [42, 43]. Furthermore, BBR increased the ratios of anti-inflammatory/proinflammatory cytokines, such as IL-10/IL-6, IL-10/IL-1*β*, and IL-10/TNF-*α* [43].

### 3.2. *M. charantia*

*M. charantia*, also known as bitter melon, has been used for centuries in TCM as an antipyretic and alexipharmacon herb. Recent studies have shown that *M. charantia* can ameliorate oxidative stress, hyperlipidemia [44], inflammation [45], and apoptosis [46]. It also regulates glucose metabolism by acting as a “plant insulin” [47] and presents antidiabetic and antioxidant activities [48].

#### 3.2.1. Effect of *M. charantia* on Diabetes

*Momordica charantia* (*M. charantia*) is a widely used traditional remedy for diabetes. It has been proven to be beneficial to insulin resistance, prediabetes, weight losing, and glycemic control in cultured cells, animal models, and human studies [49]. *M. charantia* can repair damaged beta cells, increase insulin levels, and also enhance insulin sensitivity. It inhibits the intestinal glucose absorption by inhibiting glucosidase and enterotoxin activities. It also stimulates the synthesis and release of thyroid hormones and adiponectin and enhances the activity of AMP-activated protein kinase (AMPK) [50]. A recent study showed that compared with metformin, the application of *M. charantia* in diabetes patients had lower probability of hypoglycemia although it is less effective than metformin in lowering blood glucose [51]. In addition, *M. charantia* has a superposition effect when taken with other hypoglycemic agents at the same time, and thus, patients may achieve better management of blood glucose [52]. *M. charantia* also reduces the obesity of rats fed with a high-fat diet [53, 54].

#### 3.2.2. *M. charantia* Changes Gut Microbiota

The effect of *M. charantia* on intestinal flora and inflammation has been reported in obese rats fed with high-fat diets. A result showed that although the exact effect of *M. charantia* on intestinal flora was not yet known, the intestinal flora is considered to play an important role through which *M. charantia* improves obesity and metabolic diseases (including diabetes) and is worth the sustained attention [54]. It was reported that BLSP (a bitter melon formulation) treatment reduced the ratio of Firmicutes and Bacteroidetes in the intestinal microflora of diabetic rats, while the relative abundance of Ruminococcaceae, Bacteroides, and Ruminococcus was significantly lower in BLSP-treated rats than in diabetic rats. These demonstrate that BLSP can alter the proportion of specific intestinal microflora in diabetic rats [55].

#### 3.2.3. *M. charantia* Reduces Inflammatory Response in Diabetes Mellitus

*M. charantia* possesses antioxidant activities; it enhances the activity of superoxide dismutase, catalase, and nonprotein sulfhydryls and decreases lipid peroxidation. Moreover, *M. charantia* can inhibit the expression of proinflammatory cytokines (TNF-*α*, IL-6, and IL-10), inflammatory markers (NO, inducible nitric oxide synthase, and myeloperoxidase), and apoptotic markers (BAX and caspase-3) and upregulate Bcl-2 expression [46]. By suppressing the activation of NF-*κ*B by inhibiting NF-*κ*B alpha (I*κ*B*α*) degradation and phosphorylation of JNK/p38 mitogen-activated protein kinases (JNK/p38 MAPKs), *M. charantia* can inhibit inflammation and improve the insulin signaling pathway, thereby ameliorating insulin resistance [54]. *M. charantia* has been reported to inhibit inflammation and the development of diabetes mellitus in rats and mice [54, 56]. After weeks of treatment with *M. charantia*, fasting glucose, insulin, HOMA-IR index, serum lipid levels, fat cell size of epididymal adipose tissues, blood TNF-*α*, IL-6, and IL-10 levels, and local endotoxin levels decreased in high-fat diet-induced obese rats [54]. Further studies have shown that *M. charantia* lowers mast cell recruitment in epididymal adipose tissues (EAT) and downregulates proinflammatory cytokines monocyte chemotactic protein-1 (MCP-1), IL-6, and TNF-*α* in EAT [56].

### 3.3. Curcumin

**Figure 2:**
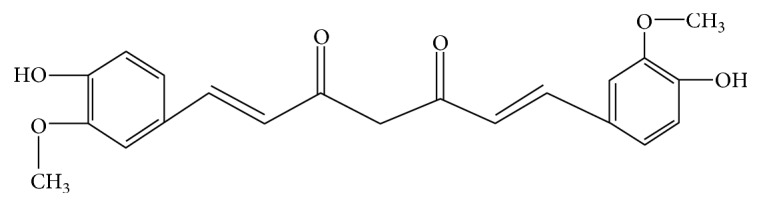
Molecular structure of curcumin.

Curcumin is the active component of rhizomes of *Curcuma longa*, a plant in the ginger family. The chemical structure of curcumin is (1E,6E)-1,7-bis(4-hydroxy-3- methoxyphenyl)hepta-1,6-diene-3,5-dione (Figure 2) [57]. Curcumin has been used as medicine for thousands of years. Recent years, extensive research on curcumin has found that curcumin has multiple biological activities, such as anticancer, anti-inflammation, antioxidation, antidiabetes, cardioprotective properties, and antiarthritis [58].

#### 3.3.1. Effect of Curcumin on Diabetes

In different experimental animal models, such as rats with alloxan-, streptozotocin- (STZ-), or STZ-nicotinamide-induced diabetes [59], oral administration of curcumin resulted in a reduction in body weight, blood glucose, and glycosylated hemoglobin levels [60] and improvement of insulin sensitivity [61]. In diabetic patients, curcumin treatment lowered blood levels of glycated hemoglobin (HbA1c) and fasting plasma glucose (FPG) and improved the pancreatic *β* cell function, as indicated by homeostasis model assessment-*β* (HOMA-*β*), C-peptide, and proinsulin/insulin ratio. Besides, curcumin can reduce the outcome of inflammatory cytokine [62] and improve relevant metabolic profiles in type 2 diabetic population [63].

#### 3.3.2. Effect of Curcumin on Gut Microbiota and Mucosal Immunity

Curcumin changes the gut microbiota. Through high-throughput sequencing, at the phylum level, Spirochaetae, Tenericutes, and Elusimicrobia were decreased and Actinobacteria was increased after curcumin treatment. At the genus level, curcumin increased the abundance of *Collinsella*, *Streptococcus*, *Sutterella*, *Gemella*, *Thalassospira*, *Gordonibacter*, and *Actinomyces* [64].

Curcumin treatment can increase the protein expression of occludin and zonula occluden-1 (ZO-1), which maintain intestinal tight junction and regulate the permeability of the intestinal epithelial barrier [16, 65]. Curcumin treatment also induces differentiation of naive CD4^+^ T cells into CD4^+^CD25^+^Foxp3^+^ Tregs and IL-10-producing Tr1 cells and increases the secretion of IL-10, TGF-*β*, and retinoic acid in the intestinal lamina propria [66]. Curcumin can also stimulate the intestinal epithelial cells and innate lymphoid cells to increase the secretion of IL-25, IL-33, IL-22, and IL-17 and play a role in anti-inflammatory activity [67].

#### 3.3.3. Curcumin Reduces Inflammatory Response in Diabetes

Curcumin suppresses inflammation through complex mechanisms and multiple targets, such as inflammatory cytokines, protein kinases, and transcription factors. Inflammatory cytokines affected by curcumin include interleukins, TNF, IFN, and COX-2. Protein kinases include Janus kinase 1/2 (JAK1/2), JNK, extracellular signal-regulated kinase 1/2 (ERK1/2), IKK, and MAPK. Transcription factors include NF-*κ*B [58].

Curcumin can reduce the production of inducible nitric oxide synthase (iNOS) and cyclooxygenase- (COX-) 2 by inhibiting LPS-induced iNOS and COX-2 gene expression [68]. iNOS, as an inflammatory signaling factor, mediated inflammation by multiple pathways. Excessive expression of iNOS in cells causes the inflammation and insulin resistance of metabolic organs [69]. COX-2 is a key enzyme in the synthesis of prostaglandins, which contributes to low-grade inflammation of tissues. It was found that curcumin exhibited anti-inflammatory activity by inhibiting the JNK/NF-*κ*B activation and the gene expression of TNF-*α*, IL-10, and IL-6 [70].

### 3.4. Ginsenoside

**Figure 3:**
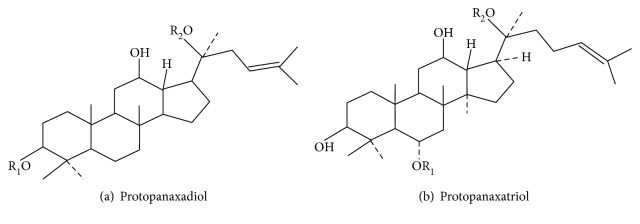
Molecular structure of ginsenoside.

*Panax ginseng* has been used for centuries in TCM as an herbal remedy. It is one of the best chosen medical plants to replenish vitality/energy, nourish body fluid, and calm the nerves [71]. The active components of ginseng have been identified as a group of saponins called ginsenosides. According to the chemical structure, ginsenosides can be divided into ginseng diol saponins, ginseng triol saponins, and oleanolic acid saponins (Figure 3). Recent studies have shown that *Panax ginseng* and its monomers have a variety of pharmacologic action such as antioxidation [72, 73], antitumor [74], anti-inflammation [75, 76], immune regulation [77], antidiabetes [78, 79], and myocardial protection [80, 81]. It can improve the immune system; inhibit inflammatory factors; protect cardiac function; lower blood glucose; inhibit rectal, liver, and breast cancers [74, 82, 83]; repair neurons; and delay the development of Parkinson's disease and Alzheimer's disease [84, 85].

#### 3.4.1. Benefits of Ginsenosides to Diabetes

Studies have shown that ginsenoside Rg1 can improve glucose and lipid metabolisms and reduce blood glucose levels and insulin resistance indices in T2DM rats [86]. Ginsenoside Ge can improve hyperglycemia by improving cholinergic and antioxidant systems in the brain of C57BL/6 mice and improve high-fat diet-induced insulin resistance, reducing triglycerides and cholesterol [72]. Peroxisome proliferator-activated receptors (PPARs) are transcription factors that play important roles in regulating glucose and lipid metabolisms and the development of atherosclerosis. A clinical study showed that ginsenosides could improve PPAR*γ* expression and lipid metabolism, thereby reducing blood glucose [87]. Another study has shown that ginsenoside Rb1 activates the insulin signaling pathway, upregulates the expression and translocation of glucose transporters in adipose tissue, and thus increases glucose uptake in adipocytes, thereby reducing blood glucose levels and improving insulin resistance [88].

#### 3.4.2. Ginsenoside Reduces the Inflammatory Response in Diabetes Mellitus

Studies have shown that inflammatory factors such as TNF-*α*, IL-6, and monocyte chemoattractant protein-1 (MCP-1) interfere with the insulin signal transduction pathway and cause insulin resistance. They can also directly damage pancreatic *β* cells [89–92]. It is reported that ginsenoside Rb1 reduces the expression of TNF-*α* and MCP-1 in 3T3-L1 cells through regulating the IKK/NF-*κ*B signaling pathway [93, 94]. In addition, ginsenoside Rb1 suppresses lipid accumulation and increases the lipolysis in 3T3-L1 adipocytes induced by TNF-*α* [94, 95]. Ginsenoside Rb1 reduced free fatty acids in the blood and fat content, improved lipid metabolism and insulin resistance, and inhibited TNF-*α*, IL-6, and other inflammatory factors in obese mice [96–99].

### 3.5. Mulberry Leaf Extract (MLE)

**Figure 4:**
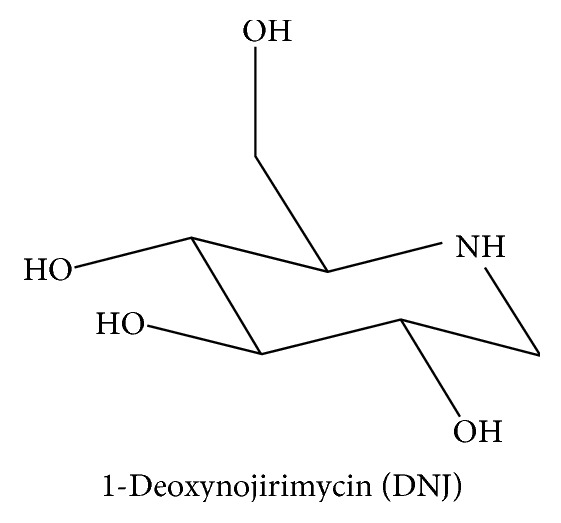
Molecular structure of DNJ.

**Figure 5:**
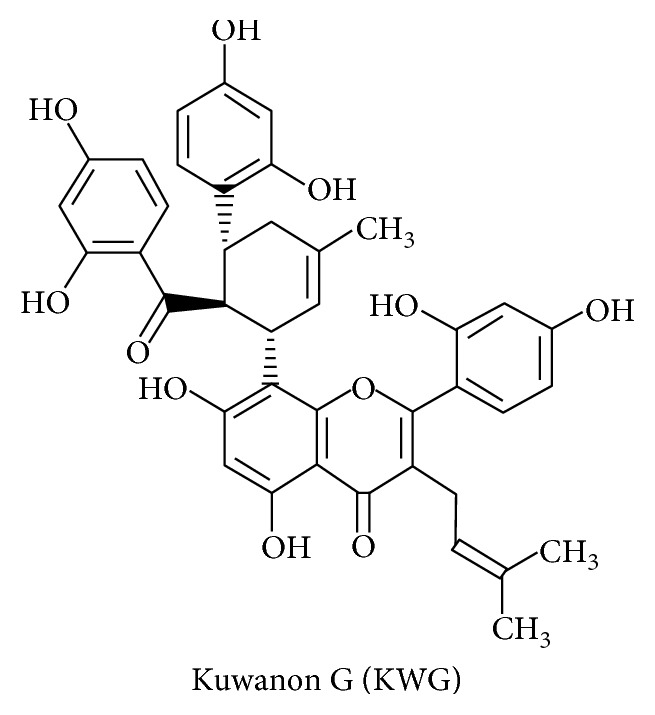
Molecular structure of KWG.

1-Deoxynojirimycin (DNJ, Figure 4) and Kuwanon G (KWG, Figure 5) are the effective constituents of mulberry leaf, which belongs to the genus *Morus* of the Moraceae family. The chemical structure of DNJ is a glucose analogue with an NH group substituting for the oxygen atom of the pyranose ring [100]. Mulberry leaf, as a traditional Chinese medicine, has been used to treat fever and inflammation for thousands of years. Recent research revealed that MLE have multiple biological activities, such as antidiabetes, antidyslipidemia, and anticancer [101].

#### 3.5.1. Benefits of MLE to Diabetes

It is reported that 1-deoxynojirimycin (DNJ) is an important component of MLE that is beneficial to the diabetes. In rats with STZ- or alloxan-induced diabetes, DNJ treatment markedly reduced blood levels of glucose and glycosylated hemoglobin and prevented the dysfunction of pancreatic *β* cells [102, 103]. A large number of studies have shown that DNJ is a competitive *α*-glucosidase inhibitor, which is present in the intestinal epithelial cells. The function of this enzyme is to hydrolyze the disaccharides to monosaccharides for absorption. DNJ inhibits the glucose absorption through competitive inhibition of *α*-glucosidase [104]. In db/db mice, DNJ treatment improved insulin resistance via the activation of the insulin signaling PI3K/AKT pathway in skeletal muscle [100] and the activation of the PKB/GSK-3*β* signaling pathway in the liver [105].

#### 3.5.2. Effects of MLE on the Intestinal Epithelial Barrier and Inflammation

Kuwanon G (KWG) which is essential in MLE is reported to protect the intestinal epithelial barrier. LPS can cause the disruption of the intestinal epithelial barrier and decrease the expression of intercellular junction protein. KWG treatment can increase the protein expression levels of occludin and intercellular adhesion molecule-1 [106].

Mulberry leaf has been used to treat fever and inflammation for thousands of years, and its extract also has anti-inflammatory effects. A study showed that MLE inhibited the expression of inflammatory cytokines IL-I, IL-6, and TNF-*α* [107] and C-reactive protein (CRP) and MLE reduced the production of iNOS [108]. The decrease in inflammatory factors is indicative of reduced chronic inflammation and results in the improvement of insulin resistance.

### 3.6. Other Herbal Monomers

In addition to the five monomers described above, many other monomers are also found to be closely related to the BMID axis, including tetrandrine [109], notoginsenoside [110], *Lycium barbarum* polysaccharide [111], allicin [112], astragaloside IV [113, 114], quercetin [115], and resveratrol [116].

Among these monomers, astragaloside IV [117], quercetin [118], and resveratrol [119] affect gut microbiota, and notoginsenoside [120], *Lycium barbarum* polysaccharide [121], and allicin [122] affect the function of alleviating intestinal mucosal immunity; the anti-inflammatory monomers are tetrandrine [123], *Lycium barbarum* polysaccharide [124], allicin [125], astragaloside IV [126, 127], quercetin [128], resveratrol [116], and the effect and possible mechanism of these monomers are summarized in Table 1.

## 4. Formulae

A formula consists of multiple Chinese herbs, which are selected under the guidance of TCM theory. In the treatment of diabetes, a formula simultaneously affects multiple targets and regulates the homeostasis, and thus, the application of formulae attracted more focus. But the mechanism research of formulae is limited. The BMID axis will provide a new perspective and make it easier for further studies.

### 4.1. Gegen Qinlian Decoction (GGQLD)

GGQLD has a very long history as a TCM formula, with the earliest record being found in the book *Treatise on Febrile and Miscellaneous Diseases* compiled by Zhongjing Zhang. GGQLD consists of extracts of Gegen (*Puerariae lobataeradix*), Huangqin (*Scutellariae radix*), Huanglian (*Coptidis rhizoma*), and Zhigancao (*Glycyrrhizae radix* et *Rhizoma Praeparata* cum Melle) [129]. In clinic, GGQLD is often used to treat ulcerative colitis and diabetes. Studies showed that GGQLD inhibits the inflammatory signaling pathway, enhances antioxidant effect, and thus improves ulcerative colitis. In addition, GGQLD improves glucose metabolism disorder, increases the insulin sensitivity index, and protects pancreatic *β* cells, playing a positive role in the treatment of diabetes [130–132].

#### 4.1.1. GGQLD Improves Diabetes

In some clinical observations and animal trials, GGQLD has been reported to have beneficial effects on diabetes. For example, in STZ- and HFD-induced diabetic SD rats, GGQLD significantly reduced FBG, HbA1c, and insulin resistance index. In 3T3-L1 adipocytes, GGQLD at 4%, 8%, and 16% was found to increase glucose consumption and decrease triglyceride in a dose-dependent manner [131]. In an observational study, T2DM patients treated with a high dose of modified GGQLD reduced blood HbA1c to 1.79% from the initial level of 9.2% [4]. Although limited, available information has demonstrated that GGQLD is beneficial to glucose metabolism and homeostasis.

#### 4.1.2. GGQLD Alters Gut Microbiota

There is an established connection between an altered gut microbiota and metabolic disorders such as obesity and diabetes [133, 134]. After treatment with GGQLD, the relative abundance of intestinal beneficial bacteria such as *Lachnospiracea incertae sedis*, *Gemmiger*, *Bifidobacterium*, and *Faecalibacterium* was significantly higher while harmful bacteria such as *F. prausnitzii*, *Alistipes*, *Pseudobutyrivibrio*, and *Parabacteroides* decreased. GGQLD increases butyrate production and protects the integrity of the mucosal barriers, thereby exerting anti-inflammatory effects which are beneficial to diabetes [135]. A study showed that GGQLD induces compositional changes in the intestinal microflora, increases beneficial bacteria, such as *Faecalibacterium* spp., and thus exerts an antidiabetic effect [135].

### 4.2. Danggui Liuhuang Decoction (DGLHD)

DGLHD is an old Chinese herbal formula which comes from the book *Lan Shi Mi Cang* written by Gao Li 741 years ago. DGLHD prescription consists of Dangui (*Angelica sinensis*), Shengdihuang (*Radix rehmanniae preparata*), Huangqin (*Radix scutellariae*), Huanglian (*Rhizoma coptidis*), Huangbo (*Cortex phellodendri*), and Huangqi (*Radix astragali*). Researches indicate that DGLHD decreases FBG and HbA1c levels and protects pancreatic *β* cells [136].

Recent studies have demonstrated that DGLHD possesses antidiabetic and immunomodulatory effects. For instance, DGLHD enhances glucose uptake in HepG2 cells, inhibits T lymphocyte proliferation, and suppresses the function of dendritic cells. After 16 weeks of treatment, DGLHD promotes insulin secretion, increases insulin sensitivity, and decreases the range of lymphocyte infiltration to inhibit insulitis as well as to protect pancreatic *β* cells in NOD mice [137].

DGLHD inhibited LPS-induced production of NO and IL-6 and the expression of iNOS and COX-2. Furthermore, DGLHD suppressed LPS-induced phosphorylation of ERK1/2 [138]. CD4^+^CD25^+^Foxp3^+^ Tregs exhibit immune regulatory activity and protect against autoimmune diabetes development. With oral intake of DGLHD, forkhead box transcription factor (Foxp3) mRNA expression in the pancreas and spleen increased. Foxp3 is essential for the differentiation and function of Tregs; thus, DGLHD increases the percentage of CD4^+^CD25^+^Foxp3^+^ Tregs in spleen lymphocytes, therefore inhibiting the low-grade inflammation in the pancreas. DGLHD also regulates the maturation and function of dendritic cells, increasing the expression of programmed death ligand-1 and decreasing the percentage of merocytic dendritic cell subset, which in turn decreases T cell-mediated inflammation and ameliorates diabetes [137].

### 4.3. Huanglian Wendan Decoction (HLWDD)

HLWDD is a Chinese herbal formula recorded in the book *Liu Yin Tiao Bian* [139]. It consists of root and rhizome of *Coptis deltoidea* C. Y. Cheng & P. K. Hsiao (family: Ranunculaceae), cortex of *Bambusa textilis* McClure (family: Poaceae), caulis of *Pinellia ternata* (Thunb.) Makino (family: Araceae), fructus of *Citrus aurantium* L. (family: Rutaceae), pericarpium of *Citrus reticulata* Blanco (family: Rutaceae), dried sclerotia of *Poria cocos* (Schw.) Wolf (family: Polyporaceae), root and rhizome of *Glycyrrhiza uralensis* Fisch (family: Leguminosae), and root and rhizome of *Zingiber officinale* Roscoe (family: Zingiberaceae). It has been used clinically to treat diabetes and its complications [140].

A recent study showed that treatment with HLWDD (6 g·kg^−1^ body weight) for 30 days increased the body weight and decreased FBG, triglycerides, and cholesterol compared with the diabetic model group. HLWDD decreases the release of proinflammatory cytokines, such as TNF-*α*, IL-6, and IL-1*β*, and inhibits the phosphorylation of IRS1 at the Ser307 and JNK signal pathway. Through these mechanisms, HLWDD inhibits inflammatory responses and thus improves the insulin signaling pathway [141].

## 5. Conclusions

Diabetes and its complications seriously affect human health and impose increasingly a heavy burden on the health care in many countries. Chinese herbs are inexpensive, less toxic, and tolerable than drugs. Therefore, they have been attracting increased attention in the field of diabetic prevention and treatment. Although clinical efficacy of Chinese herbs or herbal formulae is supported by emerging evidence, the mechanism is still lacking.

Through the analysis of a large number of studies on diabetic pathogenesis and treatment, we proposed the “Bacteria-Mucosal Immunity-Inflammation-Diabetes” (BMID) axis through which we attempted to explain how herbal monomers and formulae improve diabetes. Evidence has demonstrated that monomers and formulae improve diabetes and insulin function via multiple targets. Moreover, the majority of the current studies of TCM on diabetes were focused on inflammation and limited gut microbiota and intestinal mucosal immunity. Furthermore, most studies were aimed to a single target.

Functional food and natural health products have been of great interest to patients, doctors, and researchers for over a decade. Chinese herbs and herbal products are critical and a rich resource of information for the development of health products and precision medicine. Although increasing scientific evidence has been generated from clinical, preclinical, and in vitro studies, the information is still limited and lacks systemic evaluation. The mechanisms of action are particularly a weak area that needs an increased attention. BMID is our first attempt to integrate information and results of various studies and inspire a focus of future direction at which future studies can be conducted to support and improve it. We hope that this review will provide new perspectives and strategies for future research on Chinese herbal products for the prevention and treatment of diabetes and further product development.

## Figures and Tables

**Table 1:** Chinese herbal products based on the BMID axis.

Category	Chinese herbal products	“BMID” axis			
		Bacteria	Mucosal immunity	Inflammation	Diabetes
Monomers	Berberine	+	+	+	+
	*M. charantia* extract	+		+	+
	Curcumin	+	+	+	+
	Ginsenoside		+	+	+
	1-Deoxynojirimycin			+	+
	Kuwanon G		+		+
	Tetrandrine			+	+
	Notoginsenoside		+		+
	*Lycium barbarum* polysaccharide		+	+	+
	Allicin		+	+	+
	Astragaloside IV	+			+
	Quercetin	+		+	+
	Resveratrol	+		+	+

Formulae	GGQLD	+	+	+	+
	DGLHD		+	+	+
	HLWDD			+	+

“+” means positive effect while the “blank” means no effect. GGQLD: Gegen Qinlian Decoction; DGLHD: Danggui Liuhuang Decoction; HLWDD: Huanglian Wendan Decoction.
